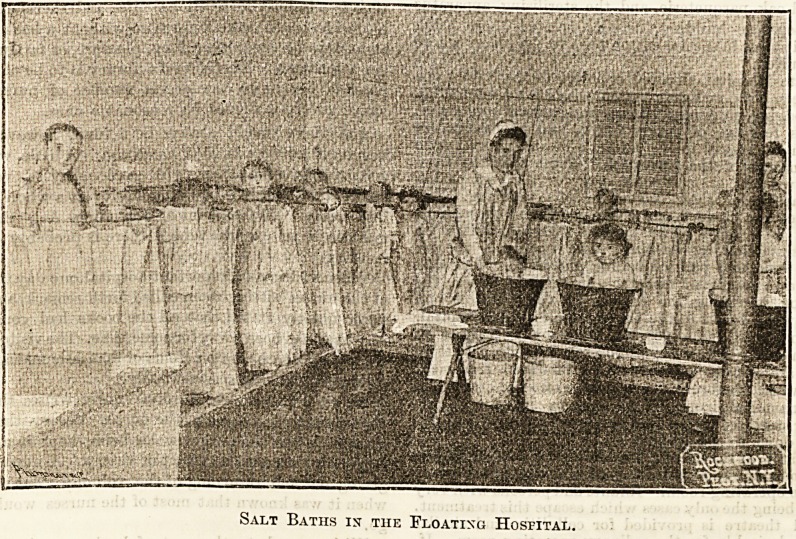# Extra Supplement.—The Nursing Mirror

**Published:** 1893-10-28

**Authors:** 


*The Hospital*, Oct. 28, 1893. *Extra Supplement.*

### "The Hospital" Nursing Mirror.

Being the Extra Nursing Supplement of "The Hospital" Newspaper.

[Contributions for this Supplement should be addressed to the Editor, The Hospital, 428, Strand, London, W.C., and should have the word "Nursing" plainly written in left-hand top corner of the envelope.]

### News from the Nursing World.

### OUR CHRISTMAS COMPETITIONS.

Each year our Christmas parcels are so warmly welcomed and appreciated by the sick poor in hospital wards that we trust every successive season will bring a steady increase in the number of garments provided for this annual distribution. The prizes will be: (1) For the best pair of socks knitted by a nurse, 5s.; (2) for the best pair knitted by any Hospital reader, 5s.; (3) for the best made flannel shirt, 10s.; (4) for the best flannel or flannelette bed-jacket, 10s.; (5) for the best flannel petticoat, 10s.; (6) for the best made and simplest shaped dressing gown made and cut out by a nurse, 20s. Long seams may be done by machine. All parcels must reach The Hospital Office, 428, Strand, in the first week of December, and they should bear the words "Needlework Competition" in left-hand corner of address label, and also the name of the sender.

### CAN NURSES BE COWARDS?

It is pitiable to hear and read of the numerous cases notified as infectious which have to remain in crowded dwellings and close rooms because there are no available beds in the fever hospitals. If one member of a family has scarlet fever, all the others must cease work and look starvation in the face, or else, by evading the law, continue to earn their daily bread and sow infection broadcast. Some of these perils could be avoided, and valuable work could be now done by nurses. Why do they shun fever hospitals? Emotional women write: "Kindly tell us where we can nurse cholera," or "Who will send me out to the lepers?" and yet the duty at their own doors is ignored. It is terrible that even now a hundred beds in the Tottenham Fever Hospital remain vacant because qualified nurses are not to be had. Either insufficient inducements for joining the staff are held out, or else nurses shirk the name of isolation; whilst they show no fear of the risks of infection faced daily in the out-patient and casualty departments of all general hospitals.

### INVALID CHILDREN.

An association with a special character of its own is one which has its headquarters at 18, Buckingham Street, Strand. Under the title of Invalid Children's Aid Association, it ought to be familiar to all our readers. Many a nurse sighs when she has to send home the little child whom she has tended back to health, for she knows it will be merged in surroundings very different to those which have secured its recovery in the hospital ward. But when it becomes the nurse's duty to give up the child, the I.C.A.A. is willing and able to keep watch over him. If the honorary secretary is addressed, he provides a visitor, and from time to time a report is given of the little one's progress, and if any relapse occurs, his return to hospital or a visit to a convalescent home is arranged for. Perhaps an invalid diet, or the loan of a perambulator, is found to meet the needs of the little patient. The work of the Association is crippled only by its limited means, and its friends gladly hail any efforts made to add to the funds. A concert has been kindly arranged for November 15th by Mrs. Hoyland, of 58, Nevern Square, S.W., from whom tickets can be obtained. Each purchaser will gladly remember that every seat paid for ensures help to an ailing or crippled little child.

### AMATEUR OR PROFESSIONAL?

"Now I will teach you a little massage," said an amateur lecturer in the course of an address on general nursing. Had she modestly offered "hints on rubbing" criticism would be unjustifiable, but massage requires much study, and could no more be thus taught than "a little violin playing" in the course of a general talk on music! Unfortunately, many women seem unable to realise that a thorough training is required for all kinds of work, and not only by the hospital nurse. Already there is a demand for women inspectors, and this certainly opens out a wide field of usefulness; but what women are duly qualified to hold such posts? In a vague sort of fashion nurses are looked to, but few of these have as yet added sanitation and a knowledge of sanitary law to the excellent foundation of practical hygiene; and without these they are not adequately prepared for positions involving peculiar responsibilities. Of course a good district nurse holding a certificate from some accredited body such as the Sanitary Institute of Great Britain and Ireland would be an ideal inspector if she possessed also tact and common sense. Mere attendance at a course of lectures and a diploma obtained from the lecturer forms no sort of adequate preparation. As well might individual class-masters and mistresses award school prizes. If women, through lack of proper preparation, are found incompetent to fill the new openings put before them, their failures will be solely their own fault. A good lesson in thoroughness is set by the regulations of the City Police, which decree that every member joining the force must attend ambulance classes until a certain amount of efficiency is attained.

### A NEW CHILDREN'S WARD.

The fine East Sussex Hospital at Hastings has long needed the addition of a children's ward, the small room for five patients hitherto used for the purpose only accommodating a small proportion of the juveniles admitted to the institution. The generosity of an anonymous donor, of Colonel Hankey, and other friends has at last provided necessary funds for the purpose, and the children's new ward is completed and opened. It contains ten beds and six cots, the furniture being presented by Mrs. Tubbs in memory of her late husband. Toys, books, and a rocking-horse have been sent by various friends, and the walls are hung with pretty pictures, which are a constant source of pleasure to the sick children. A sale of work in aid of the new ward and the general fund will be held at the hospital in November.

### NOVEL METHODS.

"How pleasant!" exclaims the visitor who finds a group of convalescent patients enjoying the change of sitting in a clean, bright day-room. But some infirmary boards devise a double use for these so-called "day" rooms, and utilise them for nurses' bed-rooms. The idea of hard-worked infirmary nurses having to sleep in such close proximity to the sick is, indeed, deplorable, but the Fulham Guardians have yet another uncommendable special practice, for they entrust the sick to assistant nurses who have had no previous experience.

### STRICTLY CONFIDENTIAL.

Certainly the proceedings at meetings sometimes fail to enhance the dignity of the guardians, and probably the fear of possible ridicule caused the Canterbury Board to take an unusual step the other day. They arranged their business so as "to get the reporters out of the way" before they considered the question of the appointment of a nurse. Surely publicity need never be shunned by any well-managed board, and the element of frivolity is always introduced by the perpetrators of weak jokes, and not by the innocent chronicler thereof. It is to be hoped that the confidential decision, which we await with some interest, will prove entirely satisfactory.

### TO WORK, NOT TO LEARN.

At a provincial hospital the education of probationers was recently discussed. "Certainly they ought to undergo some regular system of instruction, as is now the custom in most nurse-training schools," one of the staff ventured to remark. Another kindly offered to give the first course of lectures; and various comments followed, showing the obvious justice of the proposed new departure. But every plan was suddenly and forcibly arrested by a member of committee who decided with considerable asperity that he would not countenance anything of the kind as "nurses were there to work, and not to learn." It would be only common justice to have this unique sentiment printed on the forms of application sent out to would-be probationers.

### DISCHARGED CURED.

"November is the month of reflections and December of charities," said a nurse, "and these reflections being devoted to Christmas gifts it is sometimes possible to guide their course and to influence their kind." Hospitals are always impecunious, but at Christmas at any rate visitors like to individualise their donations, choosing the sick person or special ward with which to share their own blessings. If "he gives twice who gives quickly" be true, certainly he who gives what is most useful may be equally commended. Intending donors might always ask, if they do not happen to know, what is most needed. Every matron and charge nurse is ready to explain half a dozen urgent needs of her poor patients, such things as hardly come before visitors' notice. People have a general idea that patients arrive ill-clad at a hospital, but they seldom know the diffiulty of sending them out better equipped. If the life of a labourer, admitted for pneumonia, is saved by unremitting skill and care, he must not return to work without warm undergarments. If he owns none, so much the worse, not for him, but for his nurses. They do what they can, and the resident doctor probably helps, though his means too are limited, and the convalescent is sent home with but vague ideas of where his warm clothes came from. Existing Samaritan Funds by no means suffice to meet the enormous constant strain on the resources of sick wards in poor districts.

### A NEW NURSES' HOME.

The Dundee Sick Poor Nursing Society has long wanted increased accommodation for its nurses, and when Princess Louise, President of the Scottish Branch of the Queen Victoria Jubilee Institute, inspected and praised the arrangements last year she realised the necessity for a larger building. Since then a bequest of over £4,000 has permitted of the purchase of suitable premises, which, under the title of the Caird Home for Nurses, were opened last week. The ceremony was performed by Lord Reay, Vice-President of the Queen's Institute, who is well known for his interest in all plans for the advancement of nursing. Many speakers referred to the visit of Princess Louise, and to the general satisfaction with which all must regard this rapid fulfilment of the wish expressed on that occasion for proper accommodation to be provided at Dundee for an increased staff of Queen's Nurses.

### SHORT ITEMS.

Excellent work is being done at Southampton by the Queen's Nurses, but the subscriptions are by no means equal to the expenses. With such a list of patrons and patronesses this ought not to be the case. —A local doctor being asked the other day to give ambulance lectures to some enthusiastic ladies, agreed to do so on condition that an urgently needed district nurse was provided for the sick poor of the town.—At the opening by Lady Belper of the Hospital for Women at Nottingham over £640 was collected.—Mrs. Gold, the Superintendent of the sick wards at Hampstead Workhouse is retiring on a pension of £40 per annum, after eighteen years' service. She has been presented with a handsome easy chair and an address from the medical officers, the nurses, and other officials.—Miss Hamilton, M.D., has succeeded Miss Morice as physician-in-charge of the Victoria Dufferin Hospital in Calcutta.—Nurse Ayles, who has retired after twenty-one years' work in Greenwich Workhouse, received several handsome gifts and a gratifying address from the officials and inmates.—The new Nurses' Home at Blackburn Infirmary is to be opened on the 30th inst. by Mrs. Henry Harrison.—At a recent meeting in the Longfleet Schools Lady Baker, spoke in favour of the Dorset Health Association and the better nursing of the sick poor.—The St. Olave's Board of Guardians have authorised the granting of certificates to their nurses after due examination.—At the Southwell Diocesan Conference a paper was read by Dr. Lowe, followed by a discussion, on the value of trained district nurses in country parishes. It has been decided to add a wing to the Sunderland Convalescent Home at Heatherdene, Harrogate.—A very successful social evening was held at the Trained Nurses' Club, 12, Buckingham Street, Strand, on Monday, 23rd inst. A large number of members were present, and the programme of songs, music, and recitations organised by Mrs. F. R. Humphreys was greatly appreciated. This excellent club continues to follow on in the quiet and successful lines characteristic of it.—Miss Lucy Deane and Miss Rose Squire have been appointed Inspectors of Factories by the Kensington Vestry.

### On the Nursing of Diseases of the Nervous System.

### IV.—MENINGITIS.—PARAPLEGIA.

Meningitis is inflammation of the membranes or meninges of the brain. Its most common cause is tubercular disease, but it may also occur as the result of injury, and from extension of disease from the bone of the skull, middle ear disease, in blood poisoning, in syphilis, and in chronic alcoholism.

*Tubercular Meningitis* may occur in those known to be in consumption, or in those who have been previously healthy. It occurs at all ages, but is most often seen in children. Before there are any definite symptoms of cerebral disease there is often a general failure in health. The child is very irritable, has no appetite, becomes thin, and the bowels are constipated, &c. This may go on for a week or from two to four weeks; then the child may become convulsed. The attack of convulsions in some cases resembles an epileptic fit. At this stage also there is frequently vomiting, often having no relation to food. The child is feverish, irritable, and complains of the light, is sleepless, and suffers from headache. The pulse is generally quickened, but at times it is slow. This stage, which may be called the stage of irritation, lasts, as a rule, about seven days, and is followed by a drowsy condition, in which the child takes little notice of what is going on, and there may be loss of power in the limbs or paralysis of one or more of the cranial nerves. Hence the nurse must be on the look out for such symptoms; there may be squint, facial paralysis, retraction of the head, &c. A peculiar cry is sometimes uttered at this stage of the disease. It will be found also that the pulse is slow and irregular, respiration likewise is irregular, the pupils dilated and insensible to light, and the abdomen often retracted. In the final stage the child becomes comatose. Just before death convulsions frequently occur; the pulse becomes feeble, the breathing quick and irregular, and there may be evidence of more paralysis.

The time when it is difficult to diagnose tubercular meningitis is in the early stage. The diagnosis is important as meningitis is a very fatal disease. The positive symptoms are paralysis of some of the cranial nerves, an irregular pulse, and vomiting without obvious cause. In children the disease in early stages has to be distinguished from pneumonia, possibly typhoid fever, and the depressed condition following on acute diarrhœa. Meningitis occurring in a case of chronic phthisis may be thought to be delirium tremens if the patient is alcoholic. Also in adults the onset of symptoms is so obscure that people have been thought to be feigning illness when they have subsequently died of meningitis.

A patient suffering from meningitis must be in a cool, well-ventilated room, and the nurse, remembering the sensitiveness there is to noise and light, must be careful to be excessively quiet and gentle in her movements and handling of the patient. An icebag may be ordered to the head. Mercury ointment spread on a binder is often applied to the abdomen. Sucking a small piece of ice is useful in checking the vomiting. In the later stages the feeding presents difficulties.

As we are going on to consider diseases of the spinal cord, it is as well to mention in this place that meningitis may extend to the membranes of the spinal cord, or spinal meningitis may occur independently of disease of the membranes of the brain; thus it is a not very uncommon result of a deep bed-sore over the sacrum. The symptoms of spinal meningitis vary with the position and acuteness of the disease, but in the main they are the results of irritation of the nerves in the region of the inflammation and of interference with the functions of the cord, in the acute cases causing death rapidly, in the chronic cases giving rise to "compression-paraplegia."

*Paraplegia* is a form of paralysis due to disease of the spinal cord in which both sides of the body are affected, commonly to an equal extent. The amount of paralysis depends upon the position of the lesion in the spinal cord; if it only involves the cord in the lumbar region, both legs are paralysed; if it occupies the dorsal region the muscles of the abdomen are also powerless, and if it is in the cervical region the arms are paralysed as well. If there is complete destruction of any part of the cord, there is loss of sensation and of motion in all parts of the body below. But with a less degree of damage there may be complete loss of movement with little or no disturbance of sensation. Often, however, there is a complaint of pins and needles, tingling, pricking, &c. Again, there may be less damage still, so that the loss of movement may be only partial, *e.g.*, the foot may drag in walking.

A lesion occupying a small length only, in the cervical region of the spinal cord causes paralysis of all the parts below, and the extent of such paralysis would be the same however low down such a lesion might subsequently extend. The area of the spinal cord affected may to some extent be determined by the presence or absence of certain reflex movements. For if there is only a lesion for a small extent in the cord, and the part of the cord below is still active, reflex movements can be produced in that part, notwithstanding messages from the brain cannot reach it on account of the block above. On stimulating parts of the body connected with the active part of the cord, corresponding movements will be produced. Thus, if the plantar reflex can be obtained (that is drawing up the legs tickling on the soles of the feet) then the lumbar region of the cord, where the nerves to the sole come off, is all right, even though there be paralysis of all the muscles of the leg as far as the brain and will are concerned. Other reflexes are: the abdominal, that is contraction of the abdominal muscles on stroking or pinching the skin of the abdomen; the epigastric, that is contraction of the part of the rectus muscle of the abdomen, on stimulating the skin over the fourth to sixth ribs; the glutal, the scapular, &c.

There are other reflexes sometimes called "deep reflexes,' of which the common ones are the "knee-jerk" and "ankle clomes." Every nurse must have seen at some time a doctor testing the knee-jerk. The patient crosses one thigh over the other, and a smart tap given just below the knee-cap causes the foot to jerk upwards. Ankle clomes consist in rapid jerky movements of the foot obtained by rather suddenly and forcibly bending the foot so as to make the tendo Achillis tense. In order to obtain a knee-jerk the part of the cord on which it is dependent, that is, the lumbar region, must be healthy. But in health the brain controls the extent of the movement elicited in this way, and the withdrawal of this influence, as by disease of the cord above the lumbar region, will cause the jerking of the knee to be excessive when the tendon below the knee is struck. Ankle clomes is not present in health, it becomes manifest when the cerebral control is absent.

The condition of the bladder is important in paraplegia. Ordinarily, when full, it empties itself reflexly, the centre for the reflex act being in the lumbar region, but this reflex is similarly under the control of the brain, so that there is power to hinder it from taking place. If there is disease in the cord above the lumbar region, this region being healthy, but cut off from the brain, then the bladder when full empties itself automatically without the patient's knowledge. If the disease is situated in the lumbar region there is again incontinence. In cases where the catheter has to be passed by the nurse, it must be done at regular intervals, otherwise cystitis may occur from the retention of urine. Usually in paraplegic patients there is constipation, so that the derangement of defæcation is only noticed when purgatives are given.

Constant care on the part of the nurse is necessary to keep the patient's skin sound, and to secure cleanliness and comfort to the helpless sufferers. The nutrition of the skin may be affected, so that bed sores form very rapidly; hence it is essential that no parts are subject to undue pressure. Resting the heels and buttocks on pads with holes in the centre give relief, and the application of spirit and water after careful washing with soap and water is very beneficial. It will be seen that these cases are most difficult to nurse, and require an infinite amount of patience and attention to details —of bed-making, moving, lifting, &c.

### Thoughts on District Nursing.

### I.—TRAINING.

"My business is not to remake myself,
But make the absolute best of what God made."

In considering what training does, and what it does not do, there can scarcely be found a more appropriate motto than these words of one of the latest and noblest of poets. Training finds out and develops virtues and qualifications which, from want of opportunities and practice, have been hidden and unnoticed, so that very often their existence was not even suspected. It is only through patient daily routine that talents are brought to success or perfection.

But mere training will not make people what they are not by nature. Many good and clever women would never become good nurses, however much training they might undergo. They would be theoretical nurses only, not practical ones, and an unpractical nurse is like a piece of machinery which is wound up to work under certain conditions, and when anything unforeseen occurs, it breaks down, being quite unprepared to overcome the impediment. This applies to all training, whatever the art or profession studied.

The training of district nurses requires some special consideration, for this branch of the profession is often imperfectly understood, not only by the public generally, but also by nurses themselves.

There is a common idea, and a fatal one, that "any nurse" will do for district work. This impression has arisen from ignorance of what district nursing is, and therefore it will be well clearly to define this. Instead of "any nurse" doing for a district, it is necessary that a woman should be more highly trained for it than for a hospital staff nurse or "sister." The life of the poorest patient living in one of our "slums" is certainly as valuable as the life of a ward patient, or a private one in a luxurious house. With the last two classes a nurse can always obtain help; she has every appliance at hand, and the doctor will in serious cases see the patient twice or thrice daily, and can be summoned at any hour.

But in the district it is not so, the nurse rarely finds even the bare accessories of a sick room, and has nothing to work with excepting what she takes with her in her bag. She has to use her own judgment if she finds the patient worse, or if any change of symptoms has taken place since her last visit. Of course no nurse should take upon herself to prescribe or alter the doctor's treatment, but it is undoubtedly her duty to act for the benefit of her patient, and from experience of similar cases to discontinue medicine or application if she consider it advisable until the doctor's arrival.

A parish, dispensary, or club doctor is seldom to be found at home when he has once started on his round of visits, and it may be some hours before the nurse can let him know that a patient is worse. During this interval the responsibility rests upon herself. It is, therefore, most essential that district nurses should be of the highest standard of training, and of great experience, for they cannot (like hospital nurses) call instantly to their aid in a few seconds an experienced nurse or a doctor. This is only one of the many reasons why district nurses can only attain perfection by the workers employed being of the best quality.

### Nursing in South Africa.

(By a Practical Nurse.)

### THE LEPERS AT ROBBEN ISLAND.

When we started on our visit to the lepers, a little train of three trollies drawn by a horse was awaiting us. The first one filled with parcels, mails, &c.; on the second five poor lepers were placed, three men and two women; and on the last, on which was fixed a kind of seat, the visitors were crowded. The distance the last of the wards at the lunatic asylum was about a mile and a half.

We first of all came to a pretty little cottage, with pots of lilies on the "stoep." This was the Nurses' Home. Then there was a long building, which is the male hospital. We only went into one of the male wards, and found it clean and airy, in fact, one poor old man close to a door, was complaining of the draughts. Very pathetic were the little shrines, the "lares and apenates," which some had fixed beside their beds—cards, photos, shells. They received with pleasure some Christmas cards which we gave them.

The other visitors who had come on with us consisted of five or six ladies belonging to a "Sufferers' Aid Society," which collects clothes, books, toys, &c., and these are brought over every week to be distributed to the lepers. I hope the poor men are sometimes the recipients, but on that occasion everything was distributed to the women and children, who, poor things, were in ecstasies of delight at receiving a shawl, a cap, doll, or musical toy, clapping their poor maimed hands, and crowding round the visitors and their wonderful boxes. We did not see a single nurse; we introduced ourselves to the head of the female wards, who has two nurses under her, but as she was superintending the distribution of treasures, we just walked through the wards on our own account, talking to the poor patients and giving away our cards (wishing that we had brought more), and distributing fruit and sweets, &c. No one who has not witnessed it can in the least realise all that a "leper establishment" signifies; the many different forms that this terrible disease takes, disfiguring and crippling some so that they draw the blankets over their poor faces as the visitors enter; others reduced to a childish imbecility by the disease which eats away their mind as well as their body; children with bulging cheeks, noses, and chins; their heads swollen out of all proportion to their poor little bodies.

Those who have never before, even in hospital, seen such a collection of terrible sights and deformities, require some self-control to repress their horror at the sight which awaits them. It is a great mistake for visitors to call attention to some poor patient not only in a leper establishment, but in any kind of institution. Inmates should not be subjected to curious or critical remarks by strangers who seem to imagine that they are afflicted with deafness! Even lunatics comprehend remarks made about them, and very naturally resent them. As I said before, there are three hundred male lepers and a hundred and fifty to two hundred female, including a few white men and women. There are three nurses for the males, but we learnt that they were at dinner, which accounted for our not seeing them. We chartered a trolly to take us back, for with the voyage, the sea air, &c., we began to feel that lunch was desirable. Miss R. had kindly constituted herself as our hostess for the day. Instead of picnicing we sat down to a daintily spread dining-table, and in addition to our own provisions, "island" bread and fresh butter graced the board. Afterwards Miss R. gave us tea on the "stoep" where we sat and rested until, at three o'clock, the dancing for the lunatics began. We were afterwards taken by one of the nurses, a nice Irish girl, up the hill to the much frequented little chapel, and then to see a building used as a club by the inhabitants of the island, those who are neither insane nor lepers. They have a recreation society, and a very fair library, reading-room, a billiard-room, and refreshment department, where coffee and cakes are served in the evenings.

The bell sounded from the ship for re-embarkation, and so bidding a friendly good-bye, with much excitement and splashing we were carried to the boat, and thence by steamer returned to Cape Town, after spending a day not easily to be forgotten on Robben Island.

### Hospitals in Switzerland.

(By an Unprofessional Visitor.)

### LUCERNE.

At the Public Hospital in Lucerne, the medical patients are on one side, and the surgical on the other in the same wards. There are, however, seven or eight single rooms for the reception of cases which might be detrimental to the other patients, and bedding, &c., used by persons suffering from tuberculosis and other diseases undergoes complete disinfection by steam. All the floors in the Public Hospital are of well polished oak parqueterie, and the sterilising apparatus occupies a separate building, articles treated there being removed whilst hot in special cotton bags to the places where they are required.

The Private Hospital, situated opposite to the Public one contains fifteen beds, and each patient pays five francs a day for board, lodging, nursing, medicine and surgical appliances. The doctor's fees are of course extra, and operations are charged with due regard to the patient's means, as well as to the special nature of the operation. Those suffering from suppurating wounds or erysipelas, pay for the re-papering and whitewashing of their rooms, and also painting. The last charge seems a curious one as the paint used throughout the hospital is a hard white enamel specially suited to thorough disinfection by washing. The operating theatre has a mosaic floor, and painted ceiling and walls. The sink is of marble and near it is a receptacle of sterilised water; the apparatus for sterilising the dresses of the operators and bandages, towels, &c., is in the theatre itself at this hospital. The instruments all undergo the customary process of boiling, with one notable exception, for the sharp cutting instruments are merely subjected to a modified treatment with hot water before being placed on the table of glass and enamelled iron. Those persons present in the theatre wear sterilised unbleached cotton costumes with night-caps coming down to the eyebrows.

It is the custom at this hospital to administer a subcutaneous injection of morphia to a patient about fifteen minutes before he enters the operating room. Infants and persons with very weak hearts being the only cases which escape this treatment.

A second theatre is provided for cellulitis and similar diseases, not desirable for the ordinary operating room. If, however, any such patient is already isolated in a separate private ward, it is difficult for us to see what particular object is gained by taking him out of it and infecting unnecessarily any other apartment.

### Minor Appointments.

Fountain Fever Hospital.—Miss Fawcett, who trained for a year at the Victoria Hospital, Chelsea, afterwards worked for a year at the London Fever Hospital, for three years at St. Bartholomew's Hospital, and one year at the Holloway Sanatorium, Virginia Water, has been appointed Charge Nurse at the Fountain Fever Hospital.

Maidenhead Parish Nurse.—Miss H. Burkimsher, who was trained at the Sheffield General Infirmary, and afterwards worked as a district nurse in connection with the Acland Memorial Home, Oxford, has been appointed Parish Nurse at Maidenhead. Miss Burkimsher has gained excellent testimonials, and cordial good wishes accompany her to her fresh work.

### Libraries for Nurses.

(From the Nurse's Point of View.)

With regard to libraries, certain points present themselves immediately to nurses which might possibly hardly occur to those outside the profession.

If nurses are to have the full benefit of books, they must have these within easy reach. The shelves should be placed in their sitting-rooms, so that off duty time can be enjoyed and economised to the full. With regard to costly medical and standard works which need to be specially guarded from damage and loss, they may well be more watched over, and given out and returned at stated seasons to the acting librarian. "Stated seasons" being a somewhat vague term, is purposely used in this connection, for we do not sympathise with any hard and fast rule regarding the custody of books. Of course it minimises labour if a certain hour of each day be appointed for giving out volumes, but it is very absurd to establish a decree that they can be obtained only on one day of each week.

Everybody knows the difficulty of keeping a particular time free in busy institutional life, and therefore it appears inconsiderate to make a nurse go without a book for another eight days because either duty, pleasure, or fatigue prevented her attendance. Therefore if a library is to be of the greatest possible benefit to the largest number of nurses, the rules which govern it should be reasonable ones.

And there should always be a fund, however small, for keeping the books in good condition. A little watchful care and timely renovation will save many a volume from destruction, and it is bad economy to let a whole library sink into decay. The amount of rebinding, &c., then required means a large expense which there is probably difficulty in meeting.

When visiting an important hospital one day the barenness of the nurses' sitting-room called forth remark, and the Home sister quietly stated that all the books had gone to be done up; they had been away for some time. The visitors naturally wondered whether other items of domestic management were conducted there as spasmodically.

An old St. George's Hospital nurse writes that, through the interest of a chaplain, the nurses' library there was first started in her time, the books being kept in a little room, originally intended as a sort of vestry. A nurse saw to the giving out and returning of the volumes "at stated times, when it was known that most of the nurses would be free to go."

With regard to the sort of books required for nurses' libraries, it would be easy for would-be donors to convince themselves of what is needed by consulting superintendents as to what, if any, volumes are already in her possession for her staff.

Whilst discountenancing all works to which the word "doubtful" applies, it may be well to suggest that tired people specially want light, bright, clever books which will recreate without wearying brains, which, like bodies, are apt to be a little overstrained.

Books for average intellects, not for deep students, are wanted, for there is no fear that the real student will ever leave herself without "something to read."

Nurses in general study nursing handbooks and elementary medical works when they have examinations on hand; and when they want light literature they ought to have good, wholesome fiction, travels, biographies, &c. We hope for many communications from our readers on this interesting subject, and we shall be glad to hear what arrangements prevail in small institutions as well as in the better known large hospitals regarding libraries for nurses.

### United States of America.

### NOTES FROM NEW YORK CITY.—THE FLOATING HOSPITAL.

(By Our Own Correspondent.)

Nearly thirty years ago, in the City of New York, St. John's Guild was organised for the special purpose of ministering to sick infants and weary mothers, and for almost twenty years a floating hospital has been associated in the work. This charitable institution consists of a great white barge carrying 1,500 people, constructed, arranged, and equipped for this special purpose. The precious human freight is conveyed to the great bay where life-giving draughts of sea air are absorbed during at least one long summer day. Each day during the months of July and August this barge, with the Stars and Stripes, the Union Jack, and a third flag bearing the words "Floating Hospital," may be seen with its roomy decks occupied by weary mothers and sickly babes. The floating hospital is equipped with all requisites, twenty salt spray baths and hot and cold water connections throughout. The method employed to "tub" the little folks is well shown in the illustration, and the novel experience appears by no means an unwelcome one. There are other comforts besides refreshing baths on the great] barge, and a substantial dinner is provided for all, while physicians and trained nurses are always in attendance. The commissioners of the health department are constantly showing their appreciation of the work of the guild by timely aid and encouragement. Between fifty and sixty thousand sick babies, small children, and mothers are benefited during the heat. The estimated cost per trip is 250 dollars, and the money is often provided by some philanthropic citizen. Donations become yearly more frequent, and the donors are frequently present during these trips, and are thus eye-witnesses to the good accomplished. It is certainly a most practical way of giving a restful day to the weary and delicate mothers and children, for whom such a treat would be impossible unless given free, and all who know of the work must wish continued prosperity to this excellent scheme.

Salt Baths in the Floating Hospital.

### NOTES FROM PHILADELPHIA.

(By Our Own Correspondent.)

In connection with the Pennsylvania Hospital, the Training School for Nurses was organized in 1876. It commenced with about twelve pupils, and now there are thirty-two pupil nurses, and five graduates. In the spring of 1893, their first public graduation was held and a silver badge was presented to each member of the graduating class in addition to the diploma which had been gained. Since its organization the school has graduated fifty-three nurses in all. The late Stephen Girard bequeathed a sum of money which was to be devoted to the promotion and improvement of nursing at the Pennsylvania Hospital, and the legacy was much appreciated. With the increase in the nursing staff, the necessity of adequately accommodating it began to be felt. It was, therefore, with peculiar satisfaction that the erection of a proper establishment was contemplated, and in December, 1892, the new home for nurses was completed at a cost of about fifty thousand dollars. This fine building was a gift from the Misses Blanchard, in memory of their late parents. It is a commodious and elegant building, and will accommodate forty-two nurses, each in a light, airy, separate room. There is also a dining room as well as a kitchen solely for nurses. At present this latter is not in use, but they hope in the near future to put it to its original purpose. Miss Collier, the present Superintendent of Nurses is a graduate of the Pennsylvania Training School. The insane department of the Pennsylvania Hospital (familiarly known as Kirkbride's) has as yet no training school attached to it, and it is much to be desired that the care of those suffering from brain disease should be undertaken only by qualified nurses specially trained for this important duty. There has been a recent alteration in the period of time required for graduation at the Women's Medical College of Pennsylvania. It will in future take students four years to qualify. At the School of Medicine for Women, London, England, the prescribed course has recently been extended to five years, also at Edinburgh and Glasgow.

### Nursing at Kimberley.

"A FATHER" writes that he "would feel extremely obliged for any information the editor would be good enough to give him relative to the proper course to pursue for nurses wishing to join the hospital at Kimberley, South Africa. 1. Whom to apply to. 2. From whom can rules, regulations, conditions, &c., be obtained? 3. Is passage money out allowed? 4. Pay and allowances when in the country. The above constitute the main points upon which it is difficult to obtain accurate information in this country." We submitted this letter to a lady who has a full knowledge of the nursing arrangements at Kimberley, and she has kindly supplied the following information, which must prove interesting to many readers.

KIMBERLEY HOSPITAL.

1. The only person that I know of to apply to for admission into this hospital is Sister Henrietta, Sister in Charge, the Hospital, Kimberley, Griqualand West, South Africa; she will send rules, &c., to applicants.
2. I have always understood that Sister Henrietta did not undertake to pay passage money, either to Africa or back to England. It costs about £50 to £55 to reach Kimberley first class.
3. No one obtains a post as head nurse (as the ward sisters are called) without first having worked in the wards for a longer or shorter time according to capabilities.
4. Staff nurses receive, I think, £40 a year, laundry, &c., but find their own uniform, which is black, with the usual white apron.
5. There is a private nursing staff, whose salaries vary from £40 to £50.
6. The nurses' home attached to the hospital is most comfortable, each nurse being provided with a very nice little room, opening on to a verandah and garden.
7. The commissariat is very well managed, the meals well served and an unusual variety of food.
8. The night nurses' food has received special attention. They usually go to the 7. p.m. supper. At 1 a.m. a most excellent meal consisting of meat, bread and butter, pudding, and fruit is sent to each ward with tea. At 8.30 a.m. the night nurses breakfast, and before going to the home an allowance of wine is served out to each.
9. As there are no students all surgical dressings are done by the nurses. They therefore acquire unusual experience in surgical work, and are expected to be thoroughly conversant with all instruments, and able to put out, at a moment's notice, the different ones required for various operations. The residents give good lectures. I think Sister Henrietta also lectures.

### Novelties for Nurses.

THE patterns sent out by Egerton Burnet, of Wellington, Somerset, always interest nurses, but the latest package from him contains some particularly attractive materials. The Royal serges certainly merit their title, and are worthy of any wearers. There are also special qualities which are recommended for hard wear, marked at moderate prices, in any colour, if fifty yards or more be taken. The dark blues and blacks are particularly applicable to the present season, and there are greys of every shade, and of various, but always moderate, prices. Then, besides these admirable woollen goods, Egerton Burnet offers a number of washing materials suitable for hospital and private nurses, low priced, and pretty shades. Nurses' waterproof cloaks of any pattern are made to order in the tailoring department, thus ensuring to customers, both material and fit to their taste. Children's serge and other dresses are also made up in this department, and price lists and self-measurement, forms can be obtained on application.

### For Reading to the Sick.

MISUNDERSTOOD.

PEOPLE often complain bitterly that they are "misunderstood," a form of unhappiness most frequently experienced by the young, though from time to time it darkens the lives of a vast number of their elders. For instance, a thoughtless act and jesting word uttered in the innocence of the heart are misinterpreted, and vex and annoy the friend to whom they are addressed. Some of us may be naturally shy and cold, and do not show the affection we really feel for our relations; like the kitten in Mrs. Gatty's charming story, we cannot, or at least we do not, try to "purr when we are pleased, and so get the credit of being surly, morose, and hard. Another will be set down as mean and stingy, for he lives frugally, and his almsdeeds done by stealth make no noise in the world. Then we may be continually suffering pain, or want, or both consume us; we are too proud for pity, and hate to complain; we toil on and say nothing; but we cannot be cheerful, and our work is defective, while we ourselves are accused of carelessness or ill-temper and sullenness. All this is hard to bear, but we can comfort ourselves with the certainty of our Saviour's sympathy, for no one was so much misunderstood as He. When He went about preaching and doing good He was counted mad; when He carried the glad tidings of pardon and peace to the homes of sinners the Pharisees exclaimed, "Behold a gluttonous man and a wine-bibber," while His disciples supposed He was seeking an earthly kingdom, in which they hoped for distinction. He, the Son of God, at Whose very Name the devils tremble, was accused of being possessed of an evil spirit, and that His works of mercy were wrought through the power of Beelzebub their chief. Very hard and bitter must have been this constant sense of being misunderstood. As St. John said, "The Light shined in darkness, and the darkness comprehended it not." In this particular trial, then, whether it be our character, or our actions, or our motives that are misunderstood, we can fly to Christ with the certainty that He is able to feel for us. He has experienced it all in the worst and cruellest forms that can be touched by our infirmities. He will, in His love, pity and comfort. There is one thought which should humble us and keep us low. How, if while we are chafing at the slights of our fellow-creatures, we are making the same mistakes with regard to them? When our Saviour was misunderstood, He could see into the hearts of His blasphemers, and understand their every wish and motive; but we must wait to know each other, "till the mists have cleared away," which hang between brother and brother.

Yes, when the clouds have rolled by and the shadows have passed over, we shall in the glorious world beyond "know even as we are known." Great will be our surprise to find that we have mistaken others quite as often as they have mistaken us, and our love will be all the greater for our past misunderstandings. In the meantime, we will fly for comfort to Him, "Who, as suffering Man, was misunderstood, but Who, as God, understands us everyone."

### Where to Go.

CAGE BIRD Show, 27th, 28th, and 29th October, at the Westminster Aquarium.

### Wants and Workers

The Countess of Southesk has disposed of a number of the charming scrap books which she makes for the use of sick children, and will be glad of commissions for more. The proceeds are devoted to a particularly sad and deserving case.

Old toys of every description sent before December 10th would be gratefully received at St. Mary's District Nurses' Home, Plaistow, E., for a sale of cheap toys, and also for giving away at Christmas.

### Lectures to Ladies.

(By a Member of the Class.)

Recently we have had an examination on "First Aid to the Injured," and we are going in for another on "Nursing" presently, but "of that anon," as the story books say. "First aid" is the one absorbing topic at present.

For weeks the question, "How are your bones?" has been the greeting offered to one another, followed perhaps by "Can you find your subclavian artery?" or, "Do explain nerves to me?" Bones were our strong point, and our annoyance may be imagined, when entering in fear and trembling, the room appointed for the oral and practical part of our examination, and looking wildly around for our friend the skeleton, we saw no trace of him. Not even a small bone was visible, and that provoking examiner never mentioned the human framework at all.

The comprehensive pronoun, "we," includes all the lady inhabitants of Norbury, and we are all very important persons in our own eyes. Some people might say "not very aristocratic," but they had better not let us hear them, for we always take any shadow of a hint of disparagement of our birth and breeding in very bad part. Our married ladies talk of how they used to drive in their own carriages and move in the best society, in fact, were essentially *la crême de la crême*, before matrimony.

Our examination was preceded by half-a-dozen lectures, in which our own especial doctor taught us a good deal about the human frame, and how to piece it together when it became broken, or hurt, or drowned, or in any way dilapidated. In fact, it is marvellous what knowledge we now possess.

The fateful day beheld us ushered into the room that had been retained for our lectures, and we all tried to secure the best seats, fussed about draughts, the heat of the fire, or any other thing that occurred to us. Some exclaimed with energy, "Well I declare I was never so nervous in my life," &c., &c. Footsteps approaching, we quieted down as the door opened and our examiner entered. A young, good-looking man, and those who had put on their new hats for the occasion, felt very well pleased, while those who had not been so thoughtful seemed a trifle depressed.

The manner of our examination was as follows: A certain number of questions were set, to be answered in writing, and then as our names were called we went into the side room (mentioned before as bereft of the bones), and each in turn tried to answer any questions the nice-looking young man put to us. Then we performed on small boys, provided for the purpose, any bandaging the examiner demanded.

That young man might tell tales afterwards if he chose! He could relate how one good lady spoke of her "artillary" blood, whilst another calmly remarked that hers was "venimous." Perhaps, also, he might relate how an engaging young lady described the circulation of the blood. She started, of course, at the heart, and pursued the blood through the lungs, chivied it out of the arteries, hurried it through the capillaries to the veins, and finally, breathless and triumphant, she landed it all in one hand and left it there. "But," remarked the examiner blandly, "would not that be rather uncomfortable?" There was a pause; for a moment the girl was baffled, but her brow cleared as she announced, "Then I would bandage it!"

These and similar answers occupied the attention of the examiner for two whole hours, then his work and ours was over. He went off briskly to catch a train, and we returned to take tea with each other, to discuss our answers, and speculate on results.

In the course of a few weeks we were made acquainted with these, and were perfectly amazed at our success. Only twenty had failed, all the rest having passed. But I have omitted to state that the class consisted of thirty members. If any reader of this journal feels impelled to have an accident of any sort or kind, will he kindly come to Norbury, and let it occur on the public road? Thirty helpful women will pounce upon him and treat him willingly and joyfully with all the skill and knowledge acquired at six memorable lectures on "First Aid."

### Appointments.

[It is requested that successful candidates will send a copy of their applications and testimonials, with date of election, to The Editor, The Lodge, Porchester Square, W.]

Dorset County Hospital, Dorchester.—Miss Frances E. G. Ward, who was trained at the Westminster Hospital, has been appointed Matron of the Dorset County Hospital. We congratulate her on her promotion.

West Norfolk Hospital, King's Lynn.—Miss Dalley, who was trained at Cambridge, has been made Matron of this hospital. Miss Dalley has worked at the National Orthopœdic Hospital, London, and at Ardwick Green, Manchester. We wish her success in her new appointment.

Penzance Convalescent Home.—Miss Elizabeth Rimington has been appointed Matron of Mr. Bolitho's new convalescent home for men and women at Penzance. Miss Rimington was trained at the Royal Hants Hospital, Winchester, and afterwards was five years Head Nurse at Chester Infirmary. For the last four years she has been Matron of Carmarthen County Infirmary, and such extensive experience specially fit her for the duties which she has now undertaken.

City of London Lying-in Hospital.—We have great pleasure in announcing the appointment of Miss A. F. Heanley to the important post of Matron at this hospital, and we congratulate the institution on securing the services of so experienced, thoroughly-trained, and competent a lady. Miss Heanley was trained at the Royal Southern Hospital, Liverpool, and afterwards held appointments as Sister at that hospital and at the City of London Lying-in Hospital, Superintendent (outdoor) Queen Charlotte's Hospital and of Lying-in Charity, Warwick. We most cordially wish her success in the responsible position which she has been elected to fill.

### Notes and Queries.

SPECIAL NOTICE.

The contents of the Editor's Letter-box have now reached such unwieldy proportions that it has become necessary to establish a hard and fast rule regarding Answers to Correspondents. In future, all questions requiring replies will continue to be answered in this column without any fee. If an answer is required by letter, a fee of half-a-crown must be enclosed with the note containing the enquiry. We are always pleased to help our numerous correspondents to the fullest extent, and we can trust them to sympathise in the overwhelming amount of writing which makes the new rules a necessity. Every communication must be accompanied by the writer's name and address, otherwise it will receive no attention.

### Queries.

(210) *Nurse.*—Is there any hospital which would give a few months' gratuitous training to a poor woman of 45, who appears to be eminently fitted for nursing?—*Norwood.*

(211) *Medicine.*—Can you recommend a first-class book of domestic medicine? I want it for use in my family.—*H. H. G.*

(212).—Can you tell me whether the yeast test is considered uniformly reliable for registering the amount of sugar in diabetic urine?—*A. S. A.*

### Answers.

(210) *Nurse (Norwood).*—A few months' gratuitous hospital training would be not only difficult to obtain, but could not make a skilled nurse of a woman of 45, though it might be of use to herself. If she applied to a fever hospital, where workers are just now sorely needed, she might get encouragement.

(211) *Medicine (H. H. G.).*—We cannot advise you to use any medicines in your family which have not been prescribed by a qualified practitioner.

(212) *A. S. A.*—The yeast test for the quantitative analysis of sugar in the urine is generally trustworthy if Dr. William Roberts' methods be carefully carried out.

### The Book World for Women and Nurses.

[We invite Correspondence, Criticism, Enquiries, and Notes on Books likely to interest Women and Nurses. Address, Editor, THE HOSPITAL (Nurses' Book World), 428, Strand, W.C.]

MEMOIRS OF CHARLES LELAND. By CHARLES LELAND.

The book of the week is "Memoirs of Charles Leland." It is curious and instructive that the "Breitmann Ballads," by which Leland's name will live, were never appreciated by himself at their full value. He says: "They were merely written to fill up letters to various friends. I kept no copy of them—in fact, utterly forgot them. The public found them out before I did, and it's not very often that it gets ahead of a poet in appreciating his own works." Leland has an immense bump of self-esteem, and has written many more pretentious works which, however, are but of mediocre interest to the general run of both "classes and masses," while Breitmann is quoted throughout the English-speaking world. The memoirs abound with amusing stories. When slavery was abolished in the Southern States of America, one old darkey was asked why he had always refused to be liberated. He replied: 'Kase it paid. Dere's nuffin pays like bein' a dewoted darkey. De lass time I went Norf wid Massa, I made 'nuff out of him to buy myself free twice't over." Leland was dining once with Sir Charles Dilke. "There was present a small Frenchman to whom I had not been introduced. After dinner I turned over with this gentleman a curious collection of the works of Blake, which were new to him. Finding that he evidently knew something about art, I explained to him that Blake was a very strange visionary who believed that the spirits of the dead appeared to him and that he took their portraits. "*C'était donc un fou!*" (He must have been mad!) said the Frenchman. "No, Monsieur," I replied, "he was not a madman. He was almost a genius. Indeed, *c'était un Doré manqué*." (He was all but a Doré.) There was a roar of laughter from all around, and I, innocently supposing that I had said something clever unawares, laughed too. After all had departed and I was smoking alone with Sir Charles, he said, "Well, what did you think of Doré?" The little Frenchman was the great Gustave himself.

A HANDBOOK FOR MOTHERS. By JANE H. WALKER, M.D. (Longmans, Green and Co. Price 2s. 6d.)

It is with great satisfaction that we have read "A Handbook for Mothers," by Jane H. Walker, M.D. It is not such a very easy matter to avoid medical terms in giving simple and accurate accounts and plain directions, and yet this is just what this writer has accomplished. Her book contains all that is necessary for the mother to know in order to manage herself and her child well; and even the chapter on the "Disorders and Diseases of Pregnancy," which those who are perfectly well are told to omit, can hardly be said to be alarming to even a nervous person.

Throughout the book, there is a most invigorating tone, and it is written in such a pleasant, and easy style that it cannot be too strongly recommended. Women, at a time when they must turn somewhere for advice and guidance, will find this book an excellent and reliable help—far more to the purpose we venture to say than most of the personal experiences volunteered by kind-hearted but sometimes injudicious relations and friends. The irreparable harm that is done to

**THE BOOK WORLD FOR WOMEN AND NURSES**—*continued.*

children in the first years of their lives comes so largely from ignorance, that every one wishful to rear healthy and sound infants can advantageously study the simple hints and plain directions which Miss Jane Walker has so pleasantly put within every woman's reach.

The Boy God—Troublesome and Vengeful. (London: Fisher Unwin. 1893.)

Here we find in the pages of a daintily got-up little volume a series of discussions on "Love, Modern Love as Exemplified in novels," all of which discussions take the form of debates at a certain ladies' college known throughout the book as the "Camelot." This public initiation into the mysteries of a ladies' college debating society is not of so interesting a nature as to cause one's curiosity to seek any further acquaintance with it. For life intellectual at Camelot Hall savours strongly of the mental platform of boarding scools of past ages, into which has crept the assertive independent attitude of mind, which a later generation of young women have assumed towards members of the opposite sex. For "The Boy God" is nothing if it is not a sustained attempt at warfare against not "love" only, but lovers and men indiscriminately. The chief trainer of the feminine mind at this academic institution is not sparing in her share of denunciation on this subject. "Love," she urges, "should be put down on a level with the other instincts—lover's love, I mean. And instinct holds a lower place than reason, you will at once admit. Now, I ask you to imagine a whole literature devoted to the praise of an instinct. We are so used to it, that we are not sufficiently shocked at this distorted view of love—the novelist's view." . . "It is worth your while," she further adds, "to take up a novel and read into it the praises of good eating wherever you find the praises of love that will give you an altogether new appreciation of the absurdity of the modern treatment of the theme." And so on this strain is the case of "love" treated, varying in degree but not in kind, but, perhaps, of somewhat less damning a nature than are the faint praises of the opposition leader of this band of feminine philosophers. The actual plot of the narrative portion of this volume lies in the fact that after all the war which is waged throughout its pages against "The Boy God" and his emissaries, he takes his "troublesome revenge," and the fair debaters at Camelot Hall fail in their tutelage of former days. When the head governess is informed (through a third party) in the final chapter of the coming marriages of her late pupils, she writes the following characteristic commentary thereon, "Perhaps these weddings may be averted, or the consequent all but universal fatty degeneration of the moral nature may not set in; or, again, by some merciful interposition these unions may be shortened, and your friends thus restored to lives of usefulness.)' But, alas for this well-wishing congratulator, "The Boy God" had triumphed over his former assailants and his vengeance was secured.

## Figures and Tables

**Figure f1:**